# Preliminary Survey of Pathogens in the Asian Honey Bee (*Apis cerana*) in Thailand

**DOI:** 10.3390/life13020438

**Published:** 2023-02-03

**Authors:** Patcharin Phokasem, Chainarong Sinpoo, Korrawat Attasopa, Sasiprapa Krongdang, Thunyarat Chantaphanwattana, Tial C. Ling, Jeffery S. Pettis, Panuwan Chantawannakul, Veeranan Chaimanee, Terd Disayathanoowat

**Affiliations:** 1 Bee Protection Laboratory, Department of Biology, Faculty of Science, Chiang Mai University, Chiang Mai 50200, Thailand; 2 Environmental Science Research Center, Faculty of Science, Chiang Mai University, Chiang Mai 50200, Thailand; 3 Department of Entomology and Plant Pathology, Faculty of Agriculture, Chiang Mai University, Chiang Mai 50200, Thailand; 4 Faculty of Science and Social Sciences, Burapha University Sa Kaeo Campus, Sa Kaeo 27160, Thailand; 5 Pettis and Assoc, LLC, Salisbury, MD 21801, USA; 6 Department of Agro-Industrial Biotechnology, Maejo University Phrae Campus, Phrae 54140, Thailand; 7 Research Center of Microbial Diversity and Sustainable Utilization, Chiang Mai University, Chiang Mai 50200, Thailand

**Keywords:** honey bees, *Apis cerana*, LSV, ABPV, BQCV, *Nosema ceranae*, microsporidians, viruses

## Abstract

Widespread parasites, along with emerging threats, globalization, and climate change, have greatly affected honey bees’ health, leading to colony losses worldwide. In this study, we investigated the detection of biotic stressors (i.e., viruses, microsporidian, bacteria, and fungi) in *Apis cerana* by surveying the colonies across different regions of Thailand (Chiang Mai in the north, Nong Khai and Khon Kaen in the northeast, and Chumphon and Surat Thani in the south, in addition to the Samui and Pha-ngan islands). In this study, we detected ABPV, BQCV, LSV, and *Nosema ceranae* in *A. cerana* samples through RT-PCR. ABPV was only detected from the samples of Chiang Mai, whereas we found BQCV only in those from Chumphon. LSV was detected only in the samples from the Samui and Pha-ngan islands, where historically no managed bees are known. *Nosema ceranae* was found in all of the regions except for Nong Khai and Khon Kaen in northeastern Thailand. *Paenibacillus larvae* and *Ascosphaera apis* were not detected in any of the *A. cerana* samples in this survey. The phylogenetic tree analysis of the pathogens provided insights into the pathogens’ movements and their distribution ranges across different landscapes, indicating the flow of pathogens among the honey bees. Here, we describe the presence of emerging pathogens in the Asian honey bee as a valuable step in our understanding of these pathogens in terms of the decline in eastern honey bee populations.

## 1. Introduction

The Asian honey bee, *Apis cerana*, is distributed throughout Asia. These bees provide not only important pollination services for plant ecosystems, but also economic value to society due to the production of honey [1]. Based on morphological characteristics [2], *A. cerana* is divided into four groups in Thailand: northern–central Thailand, southern Thailand, Samui Island, and Phuket Island). Beekeepers in Thailand have long keeping *A. cerana* using traditional log hives for more than 1000 years, and this traditional method of beekeeping continues today [1]. It is postulated that there is a great decline in pollinator populations and/or diversity around the globe [3], and that this is influenced by various factors, including habitat loss, pesticides, climate change, and the spread of emergent pathogens, parasites, and predators [4,5].

Honey bees have been found to suffer from various pathogens. The most serious pathogens occurring in honey bees include viruses, bacteria, microsporidians, and fungi [6]. These pathogens cause diseases that bring about large damage to the beekeeping industry, which can ultimately create a great economic losses worldwide [6,7,8]. The greatest concern for honey bees’ health is nosemosis (caused by *Nosema* spp.). This disease has caused great losses in some parts of Europe [8,9]. American foulbrood (AFB), caused by *Paenibacillus larvae*, is generally found in the European honey bee; however, in some parts of Asia where beekeeping overlaps with the ecological niche of *A. cerana*, interspecific pathogen transmission from *Apis mellifera* to *A. cerana* can be observed [10,11]. The fungal honey bee pathogen *Ascosphaera apis* is a common and widespread disease that can cause a severe decline in colony production [12]. Consequently, these biotic stressors can induce considerable losses to honey bee populations.

Although *A. cerana* is often considered to have lower pathogen prevalence compared to *A. mellifera* [11], most pathogens are capable of infecting multiple hosts in natural systems [13,14]. Many emerging diseases in animals are linked to the utilization of multiple hosts [13,14]. Previous studies have identified parasites and pathogens that attack honey bee colonies and are transmitted between different host species. For instance, viral diseases that occur frequently in *A. mellifera* have been found in *A. cerana* [15,16,17]. Similar patterns have also been found in various *Bumbus* species [18] and non-*Apis* hymenopteran species [19]. The reciprocal transmission of *Varroa destructor* and *N. ceranae* between *A. cerana* and *A. mellifera* has also been found to decrease honey quality and productivity [20,21,22,23]. Chinese sacbrood virus (CSBV) is a serious threat to *A. cerana*, and it has been detected in Chinese populations of *A. mellifera* [24]. These factors have caused a great decline in *A. cerana* populations over the past few years [25]. Therefore, serious colony losses worldwide have been correlated with the movement of pests and pathogens between different host populations. 

Recently, many studies have discovered newly emergent pathogens (such as *Apis mellifera* filamentous virus and Lake Sinai Virus) in different honey bee populations [26]. Global assessment of the impacts of biotic stressors on honey bees can help in devising relevant and effective control strategies for pathogens that spread across different populations. To date, little information is known about the presence of honey bee pathogens in *A. cerana* populations. In this study, we evaluated the impacts of biotic stressors on Asian honey bees across different beekeeping sectors. The aim of this study was to assess the presence of 12 honey bee viruses: acute bee paralysis virus (ABPV), aphid lethal paralysis virus strain Brookings (ALP-Br), Big Sioux River virus (BSRV), black queen cell virus (BQCV), chronic bee paralysis virus (CBPV), deformed wing virus type A (DWV-A), deformed wing virus type B (DWV-B), Israeli acute paralysis virus (IAPV), Kashmir bee virus (KBV), sacbrood virus (SBV), slow bee paralysis virus (SBPV), and Lake Sinai virus (LSV). In addition, we aimed to examine the occurrence of four types of honey bee microsporidia (i.e., *Nosema apis*, *N. ceranae*, *N. bombi*, and *Crithidia bombi*), a fungus (i.e., *Ascosphaera apis*), and a bacterium (i.e., *Paenibacillus larvae*) in *A. cerana* collected from different regions of Thailand. We also aimed to compare the presence of pathogens in *A. cerana* between islands (the Samui and Pha-ngan islands) and mainland regions. Samui Island is in the Gulf of Thailand, approximately 35 km from the town of Surat Thani, whereas Pha-ngan Island is about 15 km from Samui Island [27]. On both islands, only native honey bees can be found. There are no records in terms of honey bee management on either island. A better understanding of *A. cerana*’s health and factors causing their colony decline and affecting their productivity in different geographic regions is a fundamental step in building beekeeping knowledge and strategies for conserving pollinator diversity via sustainable beekeeping.

## 2. Materials and Methods

### 2.1. Sample Collection

**Figure 1:**
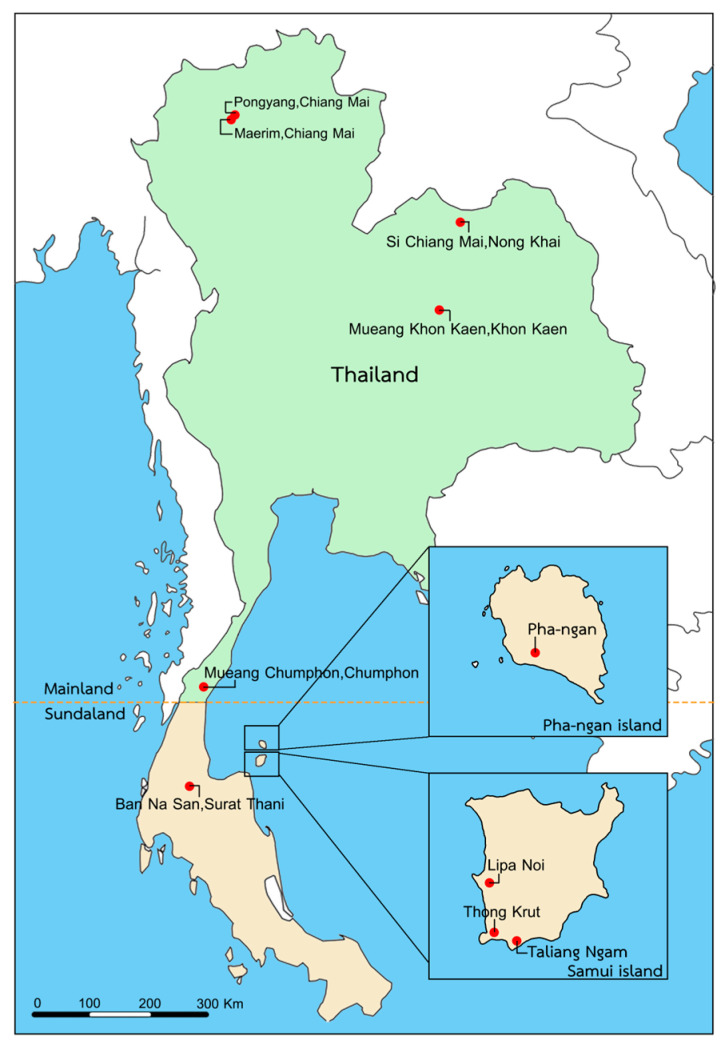
Geographic locations of the sample collection sites (red dots) of *Apis cerana* in Thailand.

The samples of adult *Apis cerana* were collected from three different regions (northern, northeastern, and southern regions) and two islands (Samui and Pha-ngan islands) in Thailand from January to June 2021. Adult of *A. cerana* samples (*n* = 50–200 for each colony) were collected from 24 colonies in 10 different locations (Figure 1 and Supplementary Table S1). All samples were preserved in RNAlater (Invitrogen, Vilnius, Lithuania) and stored at −80 °C before further examination in the laboratory.

### 2.2. DNA Extraction and DNA Analyses for Species Identification

To confirm the morphospecies identification, molecular analyses were performed using highly conserved regions of the mitochondrial cytochrome *c* oxidase subunit 1 (*COI*) gene, which is referred to as the DNA barcoding region. Genomic DNA was isolated from the whole bodies of the *A. cerana* samples (Figure 1 and Supplementary Table S1) using a DNA purification kit (PureLink Genomic DNA Mini Kit, Invitrogen, Carlsband, CA, USA) according to the manufacturer’s instructions. The primer pair was used to amplify a partial fragment DNA of the *COI* gene (listed in Table S2). The PCR amplification was performed in 25 µL reactions containing 1X PCR buffer, 1.5 mM MgCl_2_, 0.2 mM dNTPs, 0.5 µM forward primer, 0.5 µM reverse primer, 1U Taq DNA polymerase (Invitrogen, Carlsband, CA, USA), and 50 ng of DNA template. The PCR cycling conditions were 94 °C for 5 min, and 40 cycles of 94 °C for 30 s, 55 °C for 30 s, and 72 °C for 45 s, followed by a final step at 72 °C for 7 min. The resulting PCR products were separated by size on 1.5% agarose gel electrophoresis, and the nucleotide sequences were analyzed to distinguish the honey bee species. The DNA sequences were deposited in GenBank with accession numbers (see Supplementary Figure S1 and Table S3).

### 2.3. RNA Extraction and cDNA Synthesis for Viral Detection

After confirming the *A. cerana* species, 10 adult workers from each colony were pooled and homogenized using a mortar and pestle with liquid nitrogen. Total RNA was extracted using TRIzol Reagent (Invitrogen, Carlsband, CA, USA) according to the manufacturer’s instructions. The concentration of RNA was measured for absorbance at 260 nm (A260), and its purity was assessed at a ratio of A260/A280 using a BioDrop-DUO UV/Vis spectrophotometer (BioDrop, Cambridge, UK). Four micrograms of RNA was reverse-transcribed into cDNA using Tetro Reverse Transcriptase (Bioline, Memphis, TN, USA). Both oligo(dT) and random hexamer primers were used in the reaction. The mixture was incubated at 25 °C for 10 min, followed by 45 °C for 30 min, and then the reaction was terminated at 85 °C for 5 min. The cDNA was obtained and stored at −20 °C before proceeding to the next step.

### 2.4. DNA Extraction for Non-Virus Analysis

Ten adult *A. cerana* workers from each colony were pooled and homogenized as described for the initial RNA extraction step above. Total genomic DNA was extracted using a DNA purification kit (PureLink Genomic DNA Mini Kit, Invitrogen, Carlsband, CA, USA) according to the manufacturer’s instructions. DNA samples were stored at −20 °C prior to molecular screening for microsporidia, fungi, and bacteria.

### 2.5. PCR Conditions

Each sample was screened for *N. ceranae*, *N. apis*, *N. bombi*, *C. bombi*, *P. larvae*, *A. apis,* and 12 honey bee viruses; ABPV, ALP-Br, BSRV, BQCV, CBPV, DWV-A, DWV-B, IAPV, KBV, SBV, SBPV, and LSV. The specific primers used for honey bee pathogens and housekeeping genes (i.e., *β-actin* and *RPS5*) are listed in Supplementary Table S2 [28,29,30,31,32,33,34,35,36,37,38,39,40,41,42,43,44,45]. The cDNA templates were diluted 5-fold. Total DNA was diluted to 100 ng/μL. PCRs were performed using Biometra thermal cyclers (Analytik Jena AG, Jena, Germany) in 25 µL volumes containing 1 µL of DNA or cDNA template, 2.5 µL of PCR buffer, 0.75 µL of MgCl_2_, 0.5 µL of dNTPs, 0.1 µL of Taq DNA polymerase (Invitrogen, Carlsband, CA, USA), and 1.25 µL of each forward and reverse primer (10 mM), plus 17.65 µL of water. Amplification was performed with the following thermal cycling profiles: 3 min incubation at 94 °C, followed by 40 cycles of 45 s at 94 °C for denaturation, 1 min at 52–56 °C for annealing (for ABPV, IAPV, and SBPV the annealing temperature was 52 °C; for DWA-A, DWV-B, SBV, BQCV KBV, and CBPV the annealing temperature was 55 °C; for LSV, ALP-Br, BSRV, *N. ceranae*, *N. apis*, *N. bombi*, *C. bombi*, *P. larvae*, and *A. apis* the annealing temperature was 56 °C), 1 min at 72 °C for extension, and then a final step of 10 min at 72 °C. In each run, PCR mixture without DNA was used as a negative control. The amplicons obtained were electrophoresed on 1.5% agarose gel to verify the size of the fragments with reference to a 100 bp ladder (GeneDireX, Taoyuan, Taiwan). The PCR products were cleaned up using ExoSAP reagent, and then the expected amplicons were nucleotide-sequenced bidirectionally by a commercial company (Macrogen, Seoul, South Korea). 

### 2.6. Phylogenetic Tree Analysis

**Figure 2:**
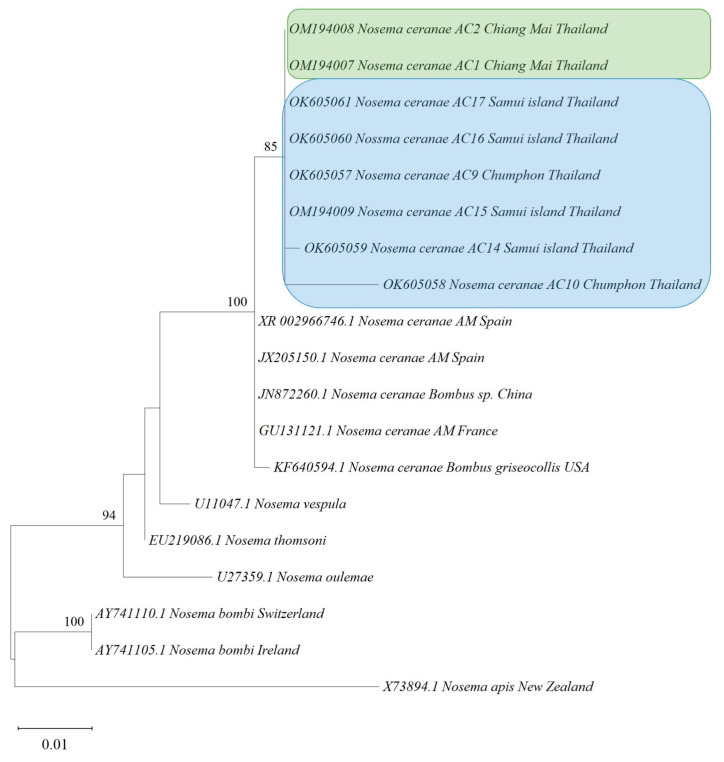
The phylogenetic tree shows the relationships of *Nosema ceranae* isolates across different countries. The partial sequences of the 16S ribosomal RNA gene of *N. ceranae* were amplified from the collected *Apis cerana*. The tree was estimated using the maximum likelihood method. The phylogenetic tree was constructed using MEGA X with 1000 bootstrap replicates. The numbers at each node represent the bootstrap values as percentages. The taxon names in the green rectangle denote the samples from the northern region. The taxon names in the blue rectangle denote the samples from the southern region. Abbreviations: AC = *Apis cerana*; AM = *Apis mellifera*.

**Figure 3:**
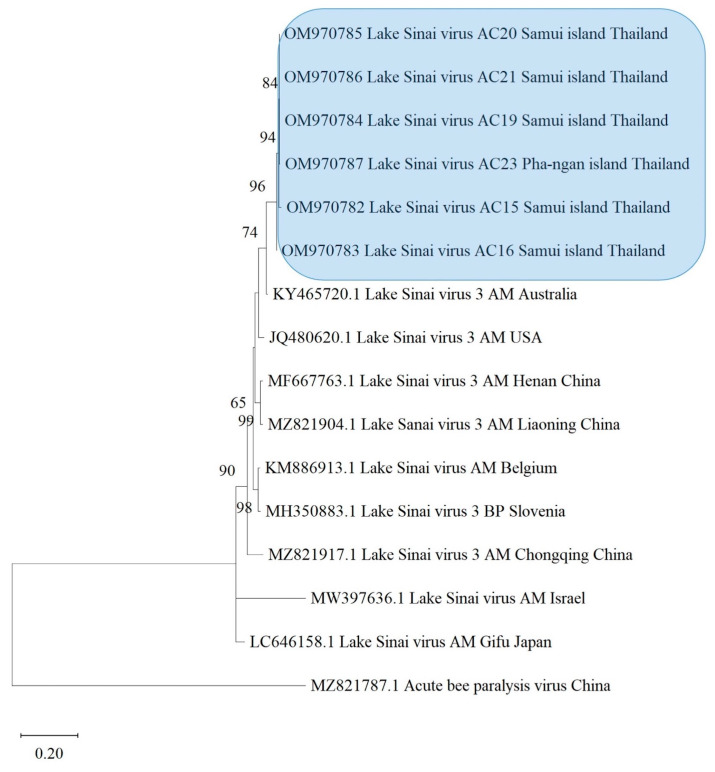
The phylogenetic tree was estimated by the maximum likelihood method based on the capsid protein coding region of LSV found in *Apis cerana* in Thailand. The phylogenetic tree was constructed using MEGA X with 1000 bootstrap replicates. The numbers at each node represent the bootstrap values as percentages. The taxon names in the blue rectangle denote the samples from the southern region. Abbreviations: AC = *Apis cerana*; AM = *Apis mellifera*; BP = *Bombus pascuorum*.

**Figure 4:**
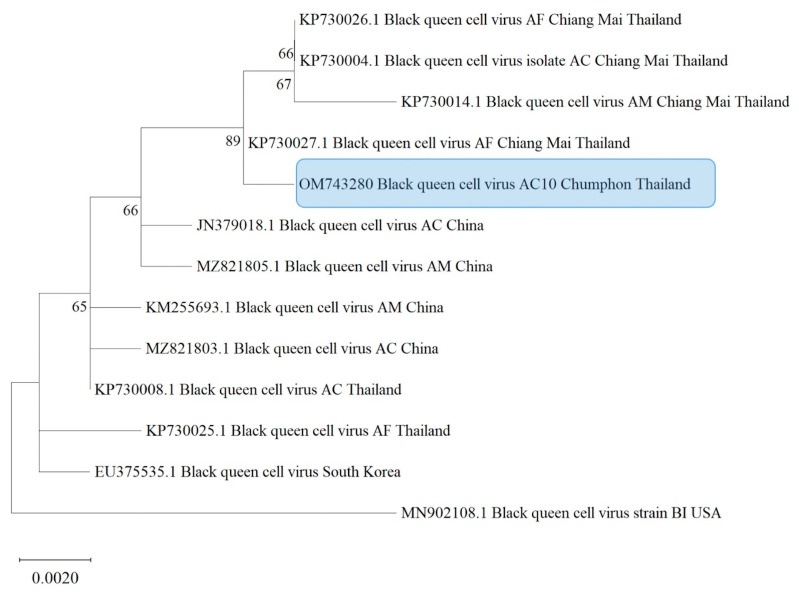
Maximum likelihood tree estimated based on the capsid protein coding region of BQCV found in *Apis cerana* in Thailand. The phylogenetic tree was constructed using MEGA X using a bootstrap value of 1000 replicates. The numbers at each node represent the bootstrap values as percentages. The taxon name in the blue rectangle denotes the samples from the southern region. Abbreviations: AC = *Apis cerana*; AM = *Apis mellifera*; AF = *Apis florea*; BI = *Bombus impatiens*.

**Figure 5:**
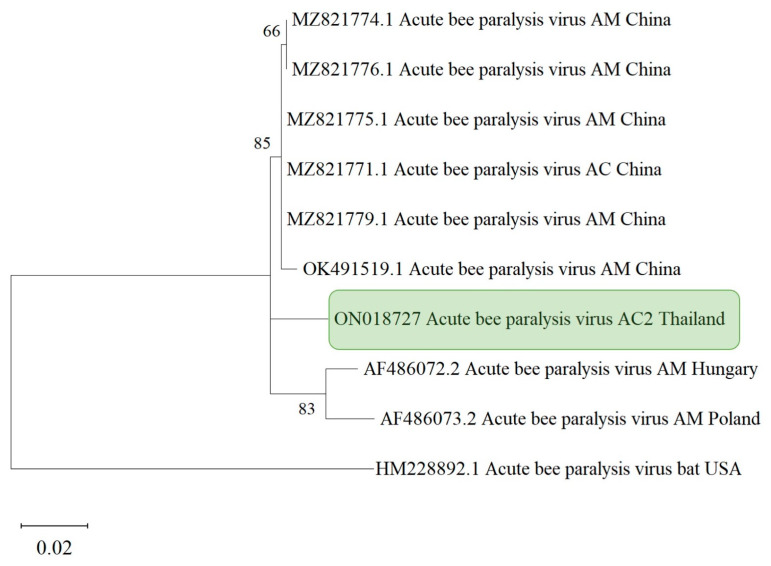
Maximum likelihood tree estimated based on the RNA-dependent RNA polymerase (RdRp) region of ABPV found in *Apis cerana* in Thailand. The phylogenetic tree was constructed using MEGA X using a bootstrap value of 1000 replicates. The numbers at each node represent the bootstrap values as percentages. The taxon name in the green rectangle denotes the samples from the northern region.

Fragment assembly of nucleotide sequences was performed using BioEdit software version 7.2.5 (Ibis Biosciences, Carlsbad, CA, USA) [46]. They were trimmed to equal size and aligned using MEGA X (iGEM, Boston, MA, USA). The obtained sequences were compared with the recorded viruses, *Nosema*, and *COI* gene in the GenBank database using the BLAST program (available from the National Center for Biotechnology Information (NCBI)). Evolutionary analyses were conducted in MEGA X [47] by using the maximum likelihood method and the Tamura–Nei model [48]. The initial tree was obtained automatically with neighbor-joining and BioNJ algorithms. The maximum likelihood trees were constructed using MEGA X. The appropriate substitution model was chosen as described in the legends of Figure 2, Figure 3, Figure 4, Figure 5 and Figure S1 for each virus, *N. ceranae*, and *COI* gene phylogeny. The bootstrap values of 1000 replicates were determined, and the percentage of replicates was shown in branches. The GenBank accession numbers from the isolates are given in the figures. The nucleotide sequences used for phylogenetic analysis were deposited in GenBank and assigned accession numbers. Related sequences of the viruses, *Nosema*, and bees used for constructing the phylogenetic trees were analyzed along with this study’s sequences (see Supplementary Figure S1 and Table S3).

## 3. Results

### 3.1. COI-Sequence-Based Characterization of A. cerana Samples

The bee phylogenetic tree based on the *COI* gene fragment estimated by the maximum likelihood method is presented in Figure S1. The tree implied the phylogenetic results of *A. cerana* subspecies appearing in Thailand. The samples from the northern and northeastern regions were identified as *A. cerana indica*, which were clustered with the same branch of *A. cerana* from India. The *A. cerana* samples from the southern regions and the Samui and Pha-ngan islands were found to be closely related to *Apis nuluensis*.

### 3.2. N. ceranae, P. larvae, and A. apis Frequencies in A. cerana Colonies

The results of *N. ceranae* prevalence are summarized in Table 1. *N. ceranae*-infected honey bees were detected among *A. cerana* workers from eight samples (33.33%). The average infection rate of *N. ceranae* in the southern region was 37.50%, while the average infection rate of *N. ceranae* in the northern region was 50.00%. Among southern locations with detectable *N. ceranae*, the highest prevalence for *N. ceranae* was found in Samui Island, with an infection rate of 25.00%. *Nosema apis*, *N. bombi*, *C. bombi*, *P. larvae*, and *A. apis* were not detected in any of the examined samples (Table 1).

### 3.3. Virus Frequencies in A. cerana Colonies

Prevalence data for 12 honey bee viruses (ABPV, ALP-Br, BSRV, BQCV, CBPV, DWV-A, DWV-B, IAPV, KBV, SBV, SBPV, and LSV) screened in 24 honey bee colonies from 10 locations are shown in Table 1. Honey bee viruses were detected in eight pools of 24 honey bee colonies. Of these eight positive pools, 29.17% of the viruses were collected in southern Thailand, followed by northern Thailand (4.17%). However, no positive viruses were found in the northeastern region. Of these viruses, only ABPV, BQCV, and LSV were detected in *A. cerana*. ABPV was found in one sample of *A. cerana* from the northern region (25.00%, Chiang Mai province). BQCV was found in one sample of *A. cerana* from the southern region (6.25%, Chumphon province). LSV was detected in six *A. cerana* samples from the southern region (37.50%, Samui and Pha-ngan islands).

### 3.4. Phylogenetic Analysis

To study the genetic relationships and variability of the studied pathogens, the nucleotide sequences of pathogens were selected from the GenBank database, and the phylogenetic analysis was carried out via maximum likelihood (ML) estimation. The clustering pattern was constructed to assess the relationships between the samples of *N. ceranae*, ABPV, BQCV, and LSV from different geographic locations. 

*Nosema ceranae* was only found in *A. cerana* from northern and southern Thailand. Based on closely related sequences of *N. ceranae* obtained from *A. cerana*, the phylogenetic tree was constructed. The *N. ceranae* isolated from northern and southern Thailand were part of the same cluster, which was different from the *N. ceranae* strain circulating in *A. mellifera* (Figure 2). However, the other cluster was *N. ceranae* detected in *A. mellifera* collected from Spain, China, France, and USA. The tree suggested that the epidemic of *N. ceranae* displayed some consistency across species between Asian and European honey bees.

LSV was found to be the most prevalent virus in *A. cerana* and was predominantly present in *A. cerana* from the southern region. The infection percentage of LSV accounted for 25.00% and affected the majority of *A. cerana* colonies. The phylogenetic tree of LSV was constructed from six isolates from *A. cerana* samples taken in this study. This also demonstrated that LSV has a genetic relationship according to host species isolation. LSV isolates from *A. cerana* colonies were clustered together in the phylogenetic tree, and they were collected from the southern region of Thailand, as well as the Samui and Pha-ngan islands. They were also nearly identical to LSV-3 isolated from *A. mellifera* from Australia, rather than other Asian isolates (Figure 3). 

According to the phylogenetic trees based on the partial sequences of the capsid protein of BQCV, two clusters were shown according to the geographic locations (Figure 4). One unique group was formed by Asian countries, including Thailand, China, and South Korea, while another group was formed by USA isolates. The USA isolates obtained from *Bombus impatiens* formed a separate cluster. BQCV was randomly present among *A. cerana*, *A. mellifera*, and *A. florea* in the Asian cluster. The spread of BQCV also appeared in all regions, and the BQCV from Asian isolates was closely related to isolates from the USA. According to the BQCV tree, the isolates obtained from *A. mellifera*, *A. cerana*, and *A. florea* indicated that the Asian BQCV cluster (Thailand, China, South Korea) may be related to their sister taxon of BQCV isolated from *Bombus impatiens* (Figure 4). 

According to the phylogenetic tree based on the RNA-dependent RNA polymerase region (RdRp) of ABPV, the sequences also formed distinct groups based on their geographical origins, regardless of honey bee species (Figure 5). Two clusters were formed: one comprising isolates from China, and one from the western isolates (i.e., Hungary and Poland). The phylogenetic tree of the ABPV isolates demonstrated that ABPV isolated from *A. cerana* was homogeneous and nearly identical to one of two distinct ABPV lineages found in *A. mellifera* (Figure 5). 

## 4. Discussion

Our results provide an important overview of the distribution of pathogens of *A. cerana* across different regions of Thailand. A total of four pathogens (ABPV, BQCV, LSV, and *N. ceranae*) were found in adult *A. cerana* samples, even though these honey bee samples did not show any symptoms of disease. Our study showed, surprisingly, that *A. cerana* honey bees located in the Samui and Pha-ngan islands of Thailand, which were not managed by beekeepers, still had LSV infections in their colonies. 

LSV was the most prevalent pathogen in *A. cerana* samples collected from the Samui and Pha-ngan islands (the distance of the two islands from mainland Thailand is approximately 35–55 km). Surprisingly, this virus was not found in the samples collected from mainland Thailand. LSV has also been detected in hornets [49], bumblebees [50,51], *A. mellifera* [52,53], *Varroa destructor* [54], and ants [55]. In addition, the *V. destructor* mite—an ectoparasite of honey bees—is known as a vector for LSV [56]. Our results are consistent with previous reports where LSV was detected in honey bees on Norfolk Island, Australia [52]. The distance of this island is approximately 1400 km from mainland Australia [57]. LSVs are very similar to chronic bee paralysis virus (CBPV) [58]. The recently described LSV was also found to be linked to a shift in gut bacterial composition that may be a biomarker of honey bee colony loss [59]. In addition, LSV1, LSV-2, LSV3, LSV6, and LSV 7 were recently discovered as honey bee viruses in the USA [59]. Phylogenetic analysis revealed one LSV-3 lineage in *A. cerana* that is closely related to LSV-3 from *A. mellifera* in Australia. In Thailand, there is no such record of the presence of LSV in *A. mellifera* or other arthropods. Therefore, our study is the first to report the presence of LSV-3 in *A. cerana* in Thailand. Experience would dictate that we need to be concerned about honey bee pathogens that can jump between *Apis* species. 

ABPV is a common infective agent of *A. mellifera* colonies that is frequently detected in healthy colonies. This virus is one of the most serious problems in the beekeeping industry. It is assumed that this virus plays a role in causing the colony loss of *A. mellifera* across the globe [60]. In this study, ABPV was detected in only one of the *A. cerana* samples from the northern region (Chiang Mai province) in Thailand. ABPV and/or its strains KBV and IAPV were detected in Asian honey bees in South Korea, China, and Japan [15,16,61]. Previous studies have reported that ABPV was detected in *A. mellifera* in northern Thailand [62]. It is possible that ABPV from *A. mellifera* jumped to *A. cerana*. In Asia, *A. mellifera* colonies share the same habitats as *A. cerana* colonies [63]. Severe colony losses are often preceded by a rapid progression of paralysis caused by viruses of the ABPV complex [64]. According to our study, there is a great need to determine the virulence of ABPV in Asian honey bees.

BQCV (a member of the Dicistroviridae) is the most abundant of the honey bee viruses and is prevalent in covert infections of most *Apis* species, including both managed and wild species [63]. BQCV has been detected in *A. cerana* in China, South Korea, Vietnam, Japan, and Thailand [10,16,17,39,65,66]. In the current study, the prevalence of BQCV was found in only one sample among *A. cerana* samples from the southern region (Chumphon province) in Thailand. Previous findings suggested that BQCV was associated with *Nosema* in *A. mellifera* colonies in which serious clinical signs were observed [67]. Our results demonstrated the co-infection of BQCV and *N. ceranae* in the same colony of Asian honey bees located in the southern region.

Both BQCV and ABPV isolates obtained from *A. cerana* fell into the same cluster as those viruses isolated from *A. mellifera*. Previously, Sanpa and Chantawannakul [62] reported that DWV, ABPV, CBPV, KBV, and SBV were found in *A. mellifera* colonies in northern Thailand. BQCV also was detected in *A. mellifera* colonies in northern Thailand [65]. These findings could be a suitable way to explain our results of viral spillover from non-native to native honey bees. Further study is needed to determine whether the transfer of these pathogens from non-native to native honey bees could be ongoing, including interspecies transmission of parasites and a potentially crucial role of host–parasite interaction. 

The microsporidia of *Nosema* spp. are obligate intracellular parasites [68]. Two species of *Nosema* have been described as infectors of honey bees (*N. ceranae* and *N. apis*) [20]. In the past, *Nosema ceranae* was found to parasitize only Asian honey bees, while *N. apis* was found to parasitize the European honey bee [69]. *Nosema ceranae* has infected *A. mellifera* and spread worldwide, leading to a decline in populations of *N. apis* [20,70,71,72,73]. Today, *Nosema ceranae* is the most common *Nosema* found in *A. cerana* and other *Apis* species [20,74,75,76]. The detection of *N. ceranae* has been reported in *A. mellifera*, *A. cerana, A. dorsata* [74,75,77], and *Bombus* spp. [78] in northern Thailand. Moreover, this parasite has been detected in *A. cerana*, *A. florea,* and the non-native *A. mellifera* in central Thailand [79]. Our results also showed that *N. ceranae* was detected in *A. cerana* samples from Chiang Mai, Chumphon, and Samui Island, but not in those collected from the northeast region. This finding is similar to that of Suraporn et al. [80], where *Nosema* was not detected in honey bees collected from the northeastern region of Thailand. However, these findings suggest that *N. ceranae* may be widespread and common in honey bees in Thailand, and potentially elsewhere in Southeast Asia. Furthermore, phylogenetic analyses can be used to separate specific taxa of *N. ceranae* in *A. cerana* and the other *Apis* isolates, as reported by Chaimanee et al. [75].

## 5. Conclusions

This study showed that the Asian honey bee (*A. cerana*) colonies distributed across several regions of Thailand were infected with several pathogens. The most prevalent pathogen was *N. ceranae,* followed by the viruses LSV, ABPV, and BQCV, in that order. The present study also reports the molecular characterization of LSV in *A. cerana* from Samui and Pha-ngan islands. Additionally, a low prevalence of ABPV and BQCV was observed in *A. cerana*. The phylogenetic tree analysis showed that pathogens can flow between host populations across the landscapes of different islands. Understanding the patterns of pathogen distribution will aid in disease control for honey bees in the future. The goal of this study was to highlight research findings that have contributed to our understanding of *A. cerana* colony health. Further investigation is needed to understand specific pathogens’ spillover processes. Increased knowledge of pathogen spillover will have important implications for the health and conservation of native honey bee species as well as other pollinators worldwide.

## Figures and Tables

**Table 1:** Frequencies of the prevalence of four pathogens recovered from *Apis cerana* in Thailand.

Sample Code	Bacteria	Fungi	Microsporidians				Viruses											
	*P. larvae*	*A. apis*	*N. apis*	*N. ceranae*	*N. bobi*	*C. bobi*	LSV	ALPV	BSRV	ABPV	BQCV	CBPV	DWVA	DWVB	IAPV	KBV	SBV	SBPV
AC1	-	-	-	+	-	-	-	-	-	-	-	-	-	-	-	-	-	-
AC2	-	-	-	+	-	-	-	-	-	+	-	-	-	-	-	-	-	-
AC3	-	-	-	-	-	-	-	-	-	-	-	-	-	-	-	-	-	-
AC4	-	-	-	-	-	-	-	-	-	-	-	-	-	-	-	-	-	-
AC5	-	-	-	-	-	-	-	-	-	-	-	-	-	-	-	-	-	-
AC6	-	-	-	-	-	-	-	-	-	-	-	-	-	-	-	-	-	-
AC7	-	-	-	-	-	-	-	-	-	-	-	-	-	-	-	-	-	-
AC8	-	-	-	-	-	-	-	-	-	-	-	-	-	-	-	-	-	-
AC9	-	-	-	+	-	-	-	-	-	-	-	-	-	-	-	-	-	-
AC10	-	-	-	+	-	-	-	-	-	-	+	-	-	-	-	-	-	-
AC11	-	-	-	-	-	-	-	-	-	-	-	-	-	-	-	-	-	-
AC12	-	-	-	-	-	-	-	-	-	-	-	-	-	-	-	-	-	-
AC13	-	-	-	-	-	-	-	-	-	-	-	-	-	-	-	-	-	-
AC14	-	-	-	+	-	-	-	-	-	-	-	-	-	-	-	-	-	-
AC15	-	-	-	+	-	-	+	-	-	-	-	-	-	-	-	-	-	-
AC16	-	-	-	+	-	-	+	-	-	-	-	-	-	-	-	-	-	-
AC17	-	-	-	+	-	-	-	-	-	-	-	-	-	-	-	-	-	-
AC18	-	-	-	-	-	-	-	-	-	-	-	-	-	-	-	-	-	-
AC19	-	-	-	-	-	-	+	-	-	-	-	-	-	-	-	-	-	-
AC20	-	-	-	-	-	-	+	-	-	-	-	-	-	-	-	-	-	-
AC21	-	-	-	-	-	-	+	-	-	-	-	-	-	-	-	-	-	-
AC22	-	-	-	-	-	-	-	-	-	-	-	-	-	-	-	-	-	-
AC23	-	-	-	-	-	-	+	-	-	-	-	-	-	-	-	-	-	-
AC24	-	-	-	-	-	-	-	-	-	-	-	-	-	-	-	-	-	-
Percentage (%)	0%	0%	0%	33%	0%	0%	25%	0%	0%	4%	4%	0%	0%	0%	0%	0%	0%	0%

Note: + = denotes presence, whereas - = denotes absence. Collection locations: AC1-2, Maerim; AC3-4, Pongyang; AC5-7, Si Chiang Mai; AC8, Mueang Khon Kaen; AC9-10, Mueang Chumphon; AC11-12, Ban Na San; AC14-17, Thong Krut; AC18, Taliang Ngam; AC19-21, Lipa Noi; AC22-24, Pha-ngan. Abbreviations: AC = *Apis cerana*.

## Data Availability

Not applicable.

## Supplementary Materials

The following supporting information can be downloaded at: https://www.mdpi.com/article/10.3390/life13020438/s1, Table S1: Sampling locations of specimens used in the present study, including geographic coordinates, elevations, and sample codes. Table S2: Primers used for molecular amplification of pathogens and mtDNA detection in the present study. Table S3: The sequences of *Nosema* spp., LSV, BQCV, ABPV, and bee specimens in our study, including other related sequences obtained from GenBank used in our phylogenetic tree analyses, showing GenBank accession numbers. Figure S1: The phylogenetic tree based on cytochrome *c* oxidase subunit I (*COI*) sequences of *Apis cerana* collected in Thailand using maximum likelihood analysis.
