# Supplementary material for: Preliminary Survey of Pathogens in the Asian Honey Bee (Apis cerana) in Thailand
Source: Life (Basel). 2023 Feb 3;13(2):438. doi: 10.3390/life13020438 (PMC9965378; doi:10.3390/life13020438)
Supplement: Supplementary file 1 [file life-13-00438-s001.zip › life-2138119-supplementary.pdf]

Supplementary information

Title: Preliminary Survey of Pathogens in the Asian Honey Bee (*Apis cerana*) in Thailand

Patcharin Phokasem, Chainarong Sinpoo, Korrawat Attasopa, Sasiprapa Krongdang, Thunyarat Chantaphanwattana, Tial C. Ling, Jeffery S. Pettis, Panuwan Chantawannakul, Veeranan Chaimanee and Terd Disayathanoowat

Table S1. Sampling locations of specimens used in the current study including geographic coordinates, elevations, and sample codes.

No.	Region of Thailand	Location (District, Province)	Sample code	Latitude	Longitude	Elevation (Meters above sea level)
1	North	Pongyang, Chiang Mai	AC1	18°53'32"N	98°50'00"E	750
2	North	Pongyang, Chiang Mai	AC2	18°53'32"N	98°50'00"E	750
3	North	Maerim, Chiang Mai	AC3	18°54'30"N	98°56'04"E	329
4	North	Maerim, Chiang Mai	AC4	18°54'30"N	98°56'04"E	329
5	Northeast	Si Chiang Mai, Nong Khai	AC5	17°58'26"N	102°23'53"E	220
6	Northeast	Si Chiang Mai, Nong Khai	AC6	17°58'26"N	102°23'53"E	220
7	Northeast	Si Chiang Mai, Nong Khai	AC7	17°58'26"N	102°23'53"E	220
8	Northeast	Mueang Khon Kaen, Khon Kaen	AC8	16°27'26"N	102°42'59"E	178
9	South	Mueang Chumphon, Chumphon	AC9	10°27'33"N	99°07'48"E	32
10	South	Mueang Chumphon, Chumphon	AC10	10°27'33"N	99°07'48"E	32
11	South	Ban Na San, Surat Thani	AC11	8°49'29"N	99°21'53"E	58
12	South	Ban Na San, Surat Thani	AC12	8°49'29"N	99°21'53"E	58
13	South	Ban Na San, Surat Thani	AC13	8°49'29"N	99°21'53"E	58
14	South	Thong Krut, Samui island	AC14	9°25'12"N	99°57'36"E	8
15	South	Thong Krut, Samui island	AC15	9°25'15"N	99°58'34"E	8
16	South	Thong Krut, Samui island	AC16	9°25'15"N	99°58'34"E	8
17	South	Thong Krut, Samui island	AC17	9°25'15"N	99°58'34"E	8
18	South	Taliang Ngam, Samui island	AC18	9°25'15"N	99°58'34"E	11
19	South	Lipa Noi, Samui island	AC19	9°30'12"N	99°56'53"E	20
20	South	Lipa Noi, Samui island	AC20	9°30'12"N	99°56'53"E	20
21	South	Lipa Noi, Samui island	AC21	9°30'12"N	99°56'53"E	20
22	South	Pha-ngan, Pha-ngan island	AC22	9°42'46"N	100°00'24"E	12
23	South	Pha-ngan, Pha-ngan island	AC23	9°42'46"N	100°00'24"E	12
24	South	Pha-ngan, Pha-ngan island	AC24	9°42'46"N	100°00'24"E	12

Table S2. Primers used for molecular amplification of pathogens, and mtDNA detection in the current study.

Target	Primer Name	Sequence	Produce size	Reference
Ascosphaera apis	ITS primers 3-F1	TGTCTGTGCGGCTAGGTG	500	[28]
	AscoAll2	GAWCACGACGCCGTCACT		
Paenibacillus larvae	Paenib-F	CTTGTGTTTCTTTCGGGAGACGCCA	1101	[29]
	Paenib-R	TCTTAGAGTGCCCACCTCTGCG		
Nosema apis	Napis-SSU-Jf1	CCATGCATGTCTTTGACGTACTATG	325	[30]
	Napis-SSU-Jr1	GCTCACATACGTTTAAAATG		
Nosema ceranae	SSUrRNA-f1	CACCAGGTTGATTCTGCCT	540	[31]
	SSUrRNA-r1b	TGTTCGTCCAGTCAGGGTCGTCA		
Nosema bombi	Nbombi-SSU-Jf1	CCATGCATGTTTTTGAAGATTATTAT	323	[30]
	Nbombi-SSU-Jr1	CATATATTTTTAAAATATGAAACAATAA		
Crithidia bombi	Crith-F	GGAAACCACGGAATCACATAGACC	500	[17]
	Crith-R	AGGAAGCCAAGTCATCCATCGC		
LSV complex	LSVdeg-F	GCCWCGRYTGTTGGTYCCCCC	578	[32]
	LSVdeg-R	GAGGTGGCGGCGCSAGATAAAGT		
aphid lethal paralysis virus strain Brookings	ALP-Br-F-2936	AACGTCGTATGCTACGATGAACTCG	646	[33]
	ALP-Br-R-3400	GGGTTAAATTCAATTCCAGTACCACGG		
Big Sioux River virus	BSRV_4714F	RGTGCAGCTTTATGCGTTGCC	519	[33]
	BSRV_37R	CCGCTGTTGAGAATAAGGATATCCAGG		
Acute bee paralysis vi rus	ABPV-F	CTCAAGTTATACGTAAAATAGCTGGAATT	646	[34]
	ABPV-R	AACCAACCTTGCTTCCCTTTA		
Black queen cell virus	BQCV-F	TGGTCAGCTCCCACTACCTTAAAC	700	[35]
	BQCV-R	GCAACAAGAAGAAACGTAAACCAC		
Chronic bee paralysis virus	CBPV-F	AGTTGTCATGGTTAACAGGATACGAG	455	[36]
	CBPV-R	TCTAATCTTAGCACGAAAGCCGAG		
Deformed wing virus A	DWV-A-F	ATCAGCGCTTAGTGGAGGAA	702	[37]
	DWV-A-R	TCGACAATTTTCGGACATCA		
Deformed wing virus B	DWV-B-F	CATGGAAATGGGATCAAACC	722	[38]
	DWV-B-R	CTTCCAAGGGCTCATCCATA		
Israeli acute paralysis virus	IAPV-F	GAAGGTTTGGGARGCYCCAYTWTGTAT	706	[39]
	IAPV-R	TGTTTGCRTCGGCHGTWGTTCCWGCAA		
Kashmir bee virus	KBV-F	GATGAACGTCGACCTATTGA	415	[40]
	KBV-R	TGTGGGTTGGCTATGAGTCA		
Sacbrood virus	SBV-F	GCTGAGGTAGGATCTTTGCGT	824	[41]

	SBV-R	TCATCATCTTCACCATCCGA		
Slow bee paralysis virus	SBPV-F	GAGATGGATMGRCCTGAAGG	915	[42]
	SBPV-R	CATGAGCCCAKGARTGTGAA		
Housekeeping genes	RPS5-F	AATTATTTGGTCGCTGGAATTG	115	[43]
	RPS5-R	TAACGTCCAGCAGAATGTGGTA		
	β-actin-F	TTGTATGCCAACACTGTCCTTT	271	[44]
	β-actin-R	TGGCGCGATGATCTTAATTT		
Cytochrome *c* oxidase subunit I (*COI*) gene	LCO1490	GGTCAACAAATCATAAAGATATTGG	658	[45]
	HCO2198	TAAACTTCAGGGTGACCAAAAAATCA		

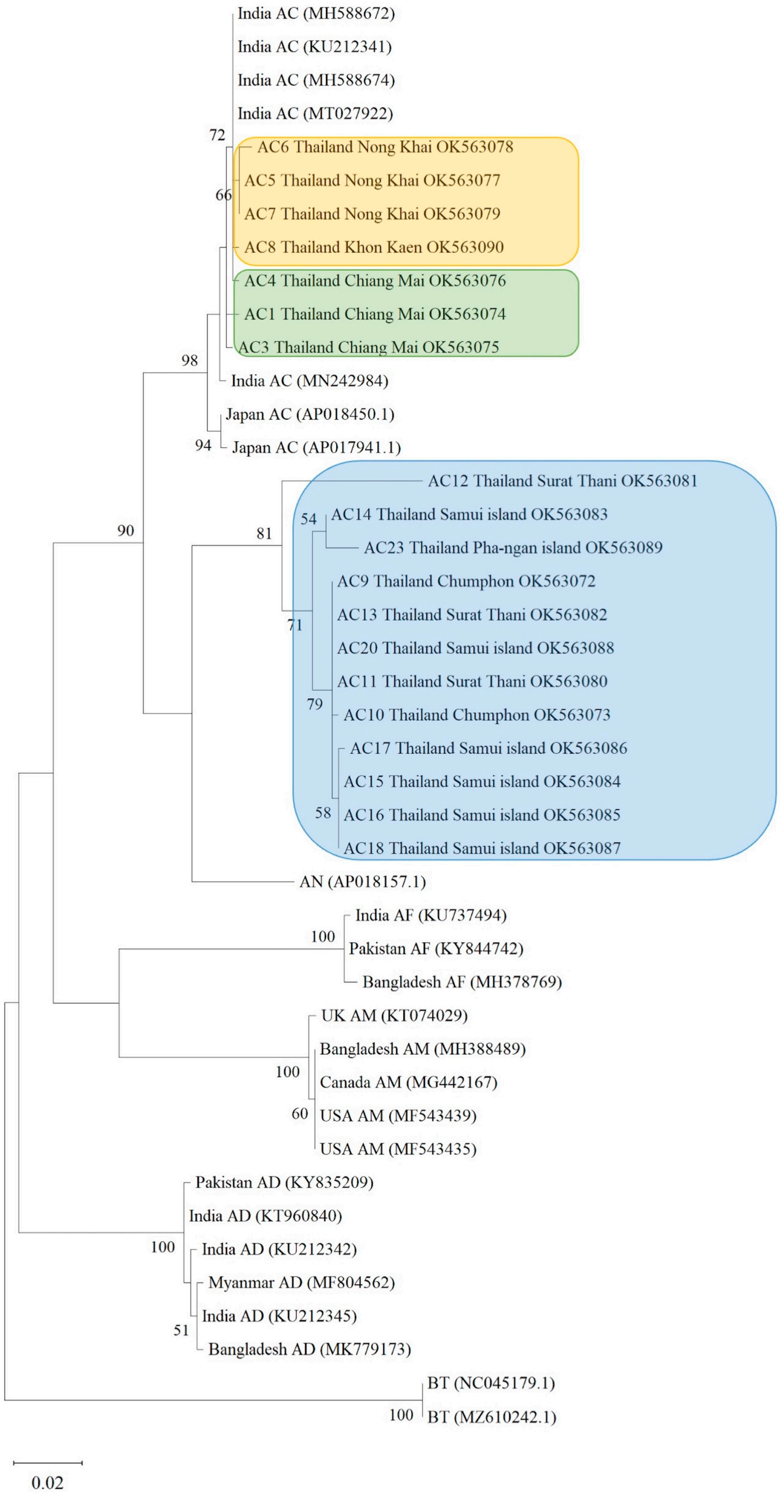

Figure S1 The phylogenetic tree based on cytochrome *c* oxidase subunit I (*COI*) sequences of *Apis cerana* collected in Thailand using maximum likelihood analysis. The sequences of *Bombus terrestris*–MZ610242.1 and NC045179.1 were used as an out group species. Numbers at each node represent bootstrap values as percentages are shown. The taxon name in yellow square denotes the sample from northeast region. The taxon name in green square denotes the sample from north region. The taxon name in blue square denotes the sample from south region. Abbreviations: AC=*Apis cerana*; AM= *Apis mellifera*; AF= *Apis florea*; AD=*Apis dorsata;* AN=*Apis nuluensis*; BT= *Bombus terrestris*.

Table S3. The sequences of *Nosema* spp., LSV, BQCV, ABPV , and bee specimens of our study including other related sequences obtained from GenBank used in our phylogenetic tree analyses showing GenBank accession numbers.

Phylogeny	Accession no.	
	This study	GenBank sequences/ references
Nosema spp.	OM194007, OM194008, OK605057, OK605059, OK605060, OK605058, OK605061, OM194009	XR002966746.1, JX205150.1, JN872260.1, X73894.1, AY741105.1, EU219086.1, U27359.1, U11047.1, GU131121.1, KF640594.1, AY741110.1
Lake Sinai Virus (LSV)	OM970785, OM970786, OM970784, OM970787, OM970782, OM970783	MF667763.1, JQ480620.1, KM886913.1, KY465720.1, LC646158.1, MH350883.1, MW397636.1, MZ821904.1, MZ821917.1, MZ821787.1
Black Queen Cell Virus (BQCV)	OM743280	KP730025.1, KP730027.1, KP730026.1, KP730014.1, KP730008.1, KP730004.1, MZ821805.1, JN379018.1, KM255693.1, MZ821803.1, EU375535.1, MN902108.1
Acute Bee Paralysis Virus (ABPV)	ON018727	AF486072.2, MZ821774.1, OK491519.1, HM228892.1, MZ821771.1, MZ821775.1, MZ821776.1, AF486073.2, MZ821779.1
Bee specimens (Cytochrome *c* oxidase subunit I (*COI*) gene)	OK563072, OK563073, OK563074, OK563075, OK563076, OK563077, OK563078, OK563079, OK563080, OK563081, OK563082, OK563083, OK563084, OK563085, OK563086, OK563087, OK563088, OK563089, OK563090	KU212345, MK779173, MF804562, KU212342, KT960840, KY835209, MT027922, MH588674, MH588672, KU212341, MN242984, MH388489, MG442167, MF543439, MF543435, KT074029, KU737494, MH378769, KY844742, AP018450.1, AP017941.4, AP018157.1, NC045179.1, MZ610242.1
